# Immunogenetic Study in Chinese Population with Ankylosing Spondylitis: Are There Specific Genes Recently Disclosed?

**DOI:** 10.1155/2013/419357

**Published:** 2013-01-16

**Authors:** Jiayu Zhai, Ju Rong, Qiuxia Li, Jieruo Gu

**Affiliations:** Department of Rheumatology and Immunology, The Third Affiliated Hospital of Sun Yat-Sen University, 600 Tianhe Road, Guangzhou, Guangdong 510630, China

## Abstract

*Purpose*. Ankylosing spondylitis (AS) is a systemic, autoimmune disease resulting in the destruction of the affected joints. Over the past 5 years, several new genes or genetic regions associated with AS have been identified in the Chinese population. This paper aims to discuss the major findings and related potential mechanisms of these studies in our population. *Recent Findings*. In recent years, due to the rapid advances in computational genetics and technology, there has been an increasing list of well-validated genes or genetic regions associated with AS susceptibility. So far, several genes or genetic regions have now been reported in the Han ethnic Chinese population, containing the major histocompatibility complex (MHC), ERAP1, IL-23R, 12q12, 2p15, 5q14.3, and so on. Different hypotheses for disease mechanisms have been investigated on the basis of the functional studies of these genes or genetic regions. *Summary*. This paper tries to summarize the association of several candidate genes with risk for AS in the Han ethnic Chinese population and aims to identify the novel inflammatory pathways and provide potential strategies for better therapies.

## 1. Introduction

Ankylosing spondylitis (AS) is a chronic, systemic, inflammatory disease that is characterized by the inflammation of the axial skeleton, the peripheral joints, the attachments of ligaments, and entheses. The main clinical feature of this disease is inflammatory low back pain, and over time some patients develop spinal immobility and ankylosis. AS appears to have multifactorial etiologies that contain underlying genetic susceptibility and environmental factors. 

In the early 1970s, the discovery of the association between HLA-B27 and AS in a sense proved that the familial aggregation of AS was genetically determined [1]. Later, more and more researches proved that HLA-B27 was not the only genetic determinant for AS. As a result of the development of genetic methodologies, from family studies with micro-satellite marker to the genome-wide association study (GWAS), the genes or genetic regions associated with AS were investigated separately. After that, many researchers investigated specific variants for particular genetic loci by a variety of approaches such as RFLP-PCR and real-time genotyping PCR. On one hand, these researchers in turn have enabled the replication of GWAS results; on the other hand, they have made those associated polymorphisms with AS to be confirmed in different populations. The aim of this paper is to discuss these findings in the Han ethnic Chinese population and what these findings provide about the pathogenesis of AS.  Table 1 shows the genes and gene regions we mentioned later. 

## 2. Epidemiology

The prevalence of AS is 0.2–0.54% among Han ethnic Chinese population which accounts for 92% of the whole population [2, 3], and it is similar to the prevalence in Europe and America [4, 5]. The clinical manifestations, severity, and risk to get the disease can vary by ethnicity, geography, and sex, with a prevalence that is higher in men during their young ages and lower in some populations such as Japan [4]. Twin studies by Brown et al. have shown that the monozygotic twin recurrence rate is 63%, compared with the dizygotic recurrence rate of 12.5% [6]. The recurrence risks in different degrees of relatives in Europeans reported by Brown were first degree relatives 8.2% (441/5390), second degree relatives 1.0% (8/834), and third degree relatives 0.7% (7/997) [7]. While, in Chinese population, the recurrence risks reported by Lin were first degree relatives 3.84% (341/8869), second degree relatives 0.87% (234/26902), and third degree relatives 0.315% (127/40258) [8]. All these data confirm that familial aggregation in AS is related to genetic factors rather than environmental ones. 

## 3. Genetic Mode

For nearly 40 years, AS has been extensively considered as a multifactorial genetic disease with a well-accredited knowledge of linkage to HLA-B27. The frequency of HLA-B27 positive status in the Han ethnic Chinese population ranged from 3.6% to 5.7% [2]. Yet, only 1–5% of these B27 positive individuals develop AS. Actually, HLA-B27 can explain no more than 30% of the overall genetic susceptibility of AS [9]. And this result suggested the contribution of additional genes or genetic regions. Meanwhile, more and more investigations supported the presence of non-MHC genetic susceptibility in this disease. Twelve years ago, Brown et al. showed us that the likely genetic model of AS was an oligogenic model with multiplicative interaction between loci, according to the study of the sibling recurrent risk [7]. Subsequently, Gu et al. initially proposed an autosomal dominant inheritance mode in a multigenerational large pedigree study of AS in the Han ethnic Chinese population based on not only pedigree investigation and segregation analysis, but also parametric linkage analysis with genome scans and fine mapping [10]. And for the first time, they reported that the incomplete penetrance of this Chinese Han pedigree was 0.54 [10]. However, it is a pity that the pathogenic genes involved have not been isolated until now. No matter which kind of inheritance mode it is, the ultimate proof of these theories is to isolate the pathogenic genes and clarify the mechanisms. 

## 4. Susceptibility Loci

Although HLA-B27 is almost essential for the inheritance of AS within families, epidemiological and genetic mode studies have suggested that there are other genetic risk factors involved. Brown et al. firstly undertook a genome-wide linkage screen in AS patients with highly polymorphic microsatellite markers and reported the susceptibility loci containing the MHC region and non-MHC region [11]. Subsequently, with genome-wide meta-analysis through aggregate analysis of four AS genome-wide scan studies, Huang and Gu managed to screen out several AS-linked loci, containing 6p22.3–p21.1, 16q, 3p, 10q, 2p, 17p, and 2q [12]. Later, by the study of the large Chinese AS families, Gu et al. validated one candidate non-MHC region for AS on 2q36.1–2q36.3 of family A and B [10]. Afterward, with the development of computational genetics and technology, especially the genome-wide association study (GWAS), some susceptibility loci such as MHC region were subsequently confirmed, and new associations of AS at 2p15 and 21q22 have been identified in a European population [13]. More recently, another GWAS in a population of Chinese descent replicated the previous associations at 2p15 and identified two additional susceptibility loci at 5q14.3 and 12q12 [14]. 

The associated SNP rs10865331 (2p15), located 99 kb upstream of B3GNT2 and 182 kb downstream of TMEM17, was found in both European and Chinese populations. However, the mechanism and function of this region still need to be explored. The associated SNP rs4552569 at 5q14.3 is located between the hyaluronan and proteoglycan link protein 1 (HAPLN1) and EGF-like repeats and discoidin I-like domains 3(EDIL3) genes. It has been reported that HAPLN1 is associated with spinal osteophyte formation and disc degeneration in Japanese women [15], while EDIL3 regulates the bone formation by way of inhibiting the Wnt signaling [16]. The association at rs17095830 of chromosome 12q12 is within an intron of anoctamin 6 (ANO6), which encodes a multipass transmembrane protein belonging to the anoctamin family. ANO6 has the Ca-dependent phospholipid scramblase activity in various biological systems and may participate in the osteoclast formation in bone [17, 18]. In the future, the validation of 5q14.3 and 12q12 in other populations and the function and mechanism investigations especially on cartilage development and bone formation should be carried on. 

## 5. MHC and HLA-B27

The major histocompatibility complex (MHC) region is a large genomic region located on chromosome 6, which is associated with disease susceptibility in most autoimmune diseases. It covers 3.6 megabases and comprises 224 known genes. Because of high allelic diversity and extreme linkage disequilibrium of MHC, the studies of MHC genes and the mechanisms that affect diseases are difficult [19]. GWAS of AS in Europe by Reveille et al. showed five highly significant associations across the MHC region (rs7743761, rs2596501, rs3915971, rs2516509, and rs1265112) [13]. Our GWAS analysis results replicated these SNPs except the rs7743761 in the Han ethnic Chinese population [14]. In addition, we reported another strong association at rs13202464, which was consistent with another GWAS analysis of AS in individuals of European ancestry [20]. 

In humans, the MHC is called the human leukocyte antigen (HLA) system, in which HLA-B27 has excessive association with AS. According to the data published in the international ImMunoGeneTics database (IMGT, release 2.24.0), 104 alleles exist, encoding 85 different proteins. All of the subtypes of HLA-B27 are ancestrally related to B*2705. Several studies have compared the intensity of the association with B*2704 and B*2705, the predominant subtypes in the Han ethnic Chinese population, and found that B*2704 was more strongly associated with AS than B*2705 [21–23]. Similarly, it has been found that B*2704 might be more strongly associated with AS than other subtypes present in Taiwanese Chinese [24]. For the Western European Caucasians, the main subtypes of B*2702 and B*2705 are equally associated with AS [22]. Other B27 subtypes are uncommon and mostly discovered in only single individuals or families. As a rare Asian subtype, B*2715 was reported in single individuals and families [25]. It is deserved to be mentioned that B*2715 was observed in AS group only and slightly more in juvenile AS group than in adult-onset AS group in our Chinese Han population study [26]. According to the above studies, it is obvious that the different B27 subtypes provide different strengths to AS, which is related to different ethnicity and geography. The different strengths associated with different B27 subtypes provide a useful method to validate the potential mechanisms of association with HLA-B27 in AS patients.

Interestingly, we have found that the association at rs13202464 remained genome-wide significant when the risk effect of HLA-B27 was controlled [14]. That is, there are possible other risk variants beyond HLA-B27 within the MHC region that are expressed by the rs13202464 [14]. Further studies are badly needed to discover the other possible MHC regions beyond B27 and investigate the mechanisms within different subtypes of B27 and the additional risk variants beyond B27.

## 6. ERAP1

In 2007, the association of ERAP1 (the gene for endoplasmic reticulum aminopeptidase 1 (ERAP-1)) with AS was first identified by the Wellcome Trust Case-Control Consortium and Australo-Anglo-American Spondyloarthritis Consortium (WTCCC/TASC) in European Caucasian population [27]. And then, the association with AS has been found in other Caucasian populations [28, 29] as well as in Chinese population [30]. Our studies demonstrated two novel SNPs (rs27434 and rs27529) in ERAP1 other than that reported previously [14]. The crystal structures in open and closed states of human ERAP1 were firstly reported by Kochan et al. [31]. ERAP1 is a zinc metallopeptidase with typical H-E-X-X-H-(X)(18)-E zinc binding and G-A-M-E-N motifs characteristic for members of the gluzincin protease family [31].

ERAP1 has three known physiological functions. First, it is involved in trimming peptides to optimal length for perfect binding and presentation by MHC class I molecules within the endoplasmic reticulum [32]. It is known to us that HLA-B27, the most important molecules in AS, belongs to MHC Class I molecules. Urgent study has investigated that the polymorphisms of ERAP1 only affect AS risk in HLA-B27 positive individuals [20]. The crystal structure study of a K528R mutant associated with AS shows significantly altered peptide processing characteristics [31]. It is proved that ERAP1 represents functionally significant interaction with specific subtypes of HLA-B27, containing B*2704 and B*2705 [33]. It is supposed that the mechanism explaining the association of HLA-B27 with AS may be related to the aberrant processing of antigenic peptides presentation associated with ERAP1. The interaction between HLA-B27 and ERAP1 should be informative suggesting the potential mechanisms. Perhaps the genetic model of AS is digenic for some individuals or families. Second, ERAP1 can promote the shedding of the proinflammatory cytokine receptors such as IL-1RII, type I TNF receptor, and the IL-6R in vitro [34]. Due to the cleavage, the inflammatory cytokines are downregulated. However, it is observed that ERAP1 does not have a major influence on cytokine receptor trimming in mice [20]. Whether the cleavage plays a role in the AS patient is still unknown. Third, ERAP1 is involved in the activation of macrophages induced by lipopolysaccharide (LPS) and interferon (IFN)-*γ* [35]. It is reported that the bacteria may be involved in the development of AS, but the mechanisms are not clarified. Researches focused on the relationship between the LPS of bacteria and ERAP1 may be informative for the mechanism investigation. 

Another report in the Spanish population showed that ERAP1 gene not only is associated with genetic predisposition to AS, but also influences the functional severity of the disease in a Spanish population [36]. The association of ERAP1 with AS in Chinese population was replicated by many researchers; however, the relation with disease severity and the potential B27-linked and -unlinked mechanisms still need to be explored. 

## 7. IL-23R

Polymorphisms in IL-23R (interleukin-23 receptor) have been recently shown to have a significant association with AS in European Caucasian population [27]. And the most strongly associated IL-23R SNPs in recent genome-wide association study were rs11209026 and rs 11465817, respectively, falling in exon9 and intron 9 of the gene [13]. Nevertheless, there was a distinguished inconsistency among the results in the Chinese Han population. Some recent studies have shown that the SNPs of IL-23R are susceptible to AS in Chinese Han population [37], while other studies have shown conflicting results [29, 38]. However, the genetic findings just provide us with a novel insight into the etiology of AS. In the future, the broader validation in different populations and specific mechanisms exploration should be carried on. 

IL-23R belongs to the hemopoietin receptor family as a subunit of the receptor for IL-23 and is involved in the production and differentiation of T helper 17(Th17) cells. Th17 cells are identified as a subset of the CD4+ T lymphocytes secreting high levels of the proinflammatory cytokine IL-17 upon stimulation [39]. In addition, related studies of Chinese population reported that the IL-23 level in the supernatants of cultured peripheral blood mononuclear cells (PBMCs) and the expression of IL-23p19 mRNA in PBMCs in patients with AS were significantly higher than that in healthy control group [40]. These investigations indirectly revealed that the polymorphisms of IL-23R were associated with AS. Interestingly, the variation of IL-23R has also been demonstrated in inflammatory bowel disease and psoriasis [41, 42], providing more or less part of the explanation as the accepted close connection between these diseases. According to all of these findings, the treatments targeting IL-23 may prove effective and need to be explored. 

## 8. Other Non-MHC Regions

Except all the non-MHC regions mentioned before, there are other regions such as anthrax toxin receptor 2 (ANTXR2), runt-related transcription factor 3 (RUNX3), interleukin-12B (IL12B), and tumor necrosis factor receptor superfamily, member 1A (TNFRSF1A) proved to be associated with AS definitively in European population [13]. The association between AS and TNFRSF1A in the Han Chinese population was confirmed by Davidson et al. several months ago [43]. At the same time, the association of IL-12B genetic polymorphism with the susceptibility of AS was proved in the Taiwanese Chinese population [44]. However, the reports about the association of RUNX3 with AS in Han Chinese population have not been found yet. Besides, based on the specific genetic loci studies, many genes such as Fc receptor-like molecule 4 (FCRL4) [45] and Fc gamma receptor IIB (FCGR2B) [46] in Han ethnic Chinese population and matrix metallopeptidase 3 (MMP-3) [47], TIMP metallopeptidase inhibitor 1 (TIMP-1) [47], and ORAI calcium release-activated calcium modulator 1 (ORAI1) [48] in Taiwanese Chinese population were reported to be associated with AS development. 

## 9. Conclusions

The development of genomic technologies such as GWAS and candidate gene studies has helped to understand the involvement of some genes in the etiology of AS. These findings strongly proved the presence of non-MHC genes involved in AS which was consistent with the prior results. However, a report showed that the familial aggregation was related to HLA-B27 other than the non-MHC susceptibility loci described recently [49]. The reason for the phenomenon needs to be explored in the future. If this can be validated in many populations, the results may be helpful for understanding the different pathogenesis of these genes. The researches concerning the genetics of AS are still in early stage. With the larger sample sizes and more new technology, more and more genes associated with AS will be identified. These new findings provide a useful platform for hypothesis-driven research into AS pathogenesis and make it possible to develop new treatments which can inhibit damage to bone structure and radiographic progression in AS.

## Supplementary Material

Genes/gene regions definitely associated with AS in Chinese population.

## Figures and Tables

**Table 1:** Genes/gene regions definitely associated with AS in Chinese population.

Chromosome location	Putative genes/candidate genes	SNP	Reference allele	Replicated or not	*P* value
6p21	HLA-B	rs13202464	G	Yes	<5.0 × 10^−324^
5q15	ERAP1	rs27434	A	Yes	6.68 × 10^−4^
5q15	ERAP1	rs30187	T	Yes	6.71 × 10^−4^
1p31	IL-23R	rs11209026	G	Yes	2.3 × 10^−17^
5q33	IL12B	rs3212227	C	Yes	/
12p13	TNFRSF1A	rs4149577	A	Yes	8.2 × 10^−4^
1q21	FCRL4	rs2777963	T	No	/
1q23	FCGR2B	rs10917661	C	No	/
12q24.31	ORAI1	rs7135617	T	No	0.008
12q24.31	ORAI1	rs712853	C	No	0.002
5q14.3	HAPLN1-EDIL3	rs4552569	C	No	8.77 × 10^−10^
12q12	ANO6	rs17095830	G	No	1.63 × 10^−8^
2p15	Unknown	rs10865331	A	Yes	1.98 × 10^−8^
6q21	Unknown	rs13210693	A	No	9.31 × 10^−7^

SNP: single nucleotide polymorphisms; HLA: human leukocyte antigen; ERAP1: endoplasmic reticulum aminopeptidase 1; IL-23R: interleukin-23 receptor; IL12B: interleukin-12B; TNFRSF1A: tumor necrosis factor receptor superfamily, member 1A; FCRL4: Fc receptor-like molecule 4; FCGR2B: Fc gamma receptor IIB; ORAI1: ORAI calcium release-activated calcium modulator 1; HAPLN1: hyaluronan and proteoglycan link protein 1; EDIL3: EGF-like repeats and discoidin I-like domains 3; ANO6: anoctamin 6.
