# Supplementary material for: Immunogenetic Study in Chinese Population with Ankylosing Spondylitis: Are There Specific Genes Recently Disclosed?
Source: Clin Dev Immunol. 2013 Jan 16;2013:419357. doi: 10.1155/2013/419357 (PMC3562651; doi:10.1155/2013/419357)
Supplement: Supplementary file 1 — Genes/gene regions definitely associated with AS in Chinese population. [file 419357.f1.doc]

Immunogenetic study in Chinese population with Ankylosing Spondylitis: Are there specific genes recently disclosed?

**Supplementary Materials**

Table 1: Genes/gene regions definitely associated with AS in Chinese population

| Chromosome location | Putative genes/ candidate genes | SNP | Reference allele | Replicated or not | P value |
| --- | --- | --- | --- | --- | --- |
| 6p21 | HLA-B | rs13202464 | G | yes | <5.0×10^−324^ |
| 5q15 | ERAP1 | rs27434 | A | yes | 6.68×10^−4^ |
| 5q15 | ERAP1 | rs30187 | T | yes | 6.71×10^−4^ |
| 1p31 | IL-23R | rs11209026 | G | yes | 2.3×10^−17^ |
| 5q33 | IL12B | rs3212227 | C | yes | / |
| 12p13 | TNFRSF1A | rs4149577 | A | yes | 8.2×10^−4^ |
| 1q21 | FCRL4 | rs2777963 | T | no | / |
| 1q23 | FCGR2B | rs10917661 | C | no | / |
| 12q24.31 | ORAI1 | rs7135617 | T | no | 0.008 |
| 12q24.31 | ORAI1 | rs712853 | C | no | 0.002 |
| 5q14.3 | HAPLN1-EDIL3 | rs4552569 | C | no | 8.77×10^−10^ |
| 12q12 | ANO6 | rs17095830 | G | no | 1.63×10^−8^ |
| 2p15 | unknown | rs10865331 | A | yes | 1.98×10^−8^ |
| 6q21 | unknown | rs13210693 | A | no | 9.31×10^−7^ |

SNP, single nucleotide polymorphisms; HLA, human leukocyte antigen; ERAP1, endoplasmic reticulum aminopeptidase 1; IL-23R, interleukin-23 receptor; IL12B, interleukin-12B; TNFRSF1A, tumor necrosis factor receptor super-family, member 1A; FCRL4, Fc receptor-like molecule 4; FCGR2B, Fc gamma receptor IIB; ORAI1, ORAI calcium release-activated calcium modulator 1; HAPLN1, hyaluronan and proteoglycan link protein 1; EDIL3, EGF-like repeats and discoidin I-like domains 3; ANO6, anoctamin 6.
